# Groundwater cable bacteria conserve energy by sulfur disproportionation

**DOI:** 10.1038/s41396-019-0554-1

**Published:** 2019-11-14

**Authors:** Hubert Müller, Sviatlana Marozava, Alexander J. Probst, Rainer U. Meckenstock

**Affiliations:** 1 Biofilm Center, University of Duisburg-Essen, Universitätsstr. 5, 45141 Essen, Germany; 2 Institute of Groundwater Ecology, Helmholtz Zentrum München, Ingolstädter Landstraße 1, 85764 Neuherberg, Germany

**Keywords:** Environmental microbiology, Microbiology

## Abstract

Cable bacteria of the family *Desulfobulbaceae* couple spatially separated sulfur oxidation and oxygen or nitrate reduction by long-distance electron transfer, which can constitute the dominant sulfur oxidation process in shallow sediments. However, it remains unknown how cells in the anoxic part of the centimeter-long filaments conserve energy. We found 16S rRNA gene sequences similar to groundwater cable bacteria in a 1-methylnaphthalene-degrading culture (1MN). Cultivation with elemental sulfur and thiosulfate with ferrihydrite or nitrate as electron acceptors resulted in a first cable bacteria enrichment culture dominated >90% by 16S rRNA sequences belonging to the *Desulfobulbaceae*. *Desulfobulbaceae*-specific fluorescence in situ hybridization (FISH) unveiled single cells and filaments of up to several hundred micrometers length to belong to the same species. The *Desulfobulbaceae* filaments also showed the distinctive cable bacteria morphology with their continuous ridge pattern as revealed by atomic force microscopy. The cable bacteria grew with nitrate as electron acceptor and elemental sulfur and thiosulfate as electron donor, but also by sulfur disproportionation when Fe(Cl)_2_ or Fe(OH)_3_ were present as sulfide scavengers. Metabolic reconstruction based on the first nearly complete genome of groundwater cable bacteria revealed the potential for sulfur disproportionation and a chemo-litho-autotrophic metabolism. The presence of different types of hydrogenases in the genome suggests that they can utilize hydrogen as alternative electron donor. Our results imply that cable bacteria not only use sulfide oxidation coupled to oxygen or nitrate reduction by LDET for energy conservation, but sulfur disproportionation might constitute the energy metabolism for cells in large parts of the cable bacterial filaments.

## Introduction

**Fig. 1:**
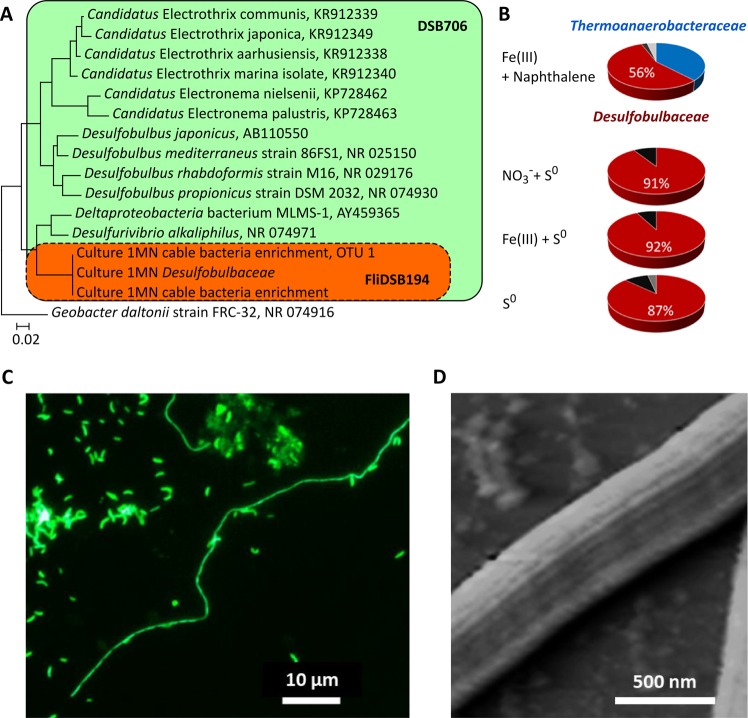
Microbial composition in the obtained enrichment cultures. **a** Maximum likelihood phylogenetic tree of full-length 16S rRNA gene sequences of *Desulfobulbaceae* retrieved from the NCBI database in comparison to the cable bacteria (MAG Dsb_1MN) from culture 1MN (red frame). Partial sequences from amplicon sequencing (OTU 1) and sequences from the metagenomes of culture 1MN (MAG Dsb_1MN) and the cable bacteria enrichment showed 100% similarity. Scale bar represents the number of substitutions per site. Known cable bacteria are represented by full-length 16S gene sequences of *Candidatus* Electrothrix and *Candidatus* Electronema. **b** Changes in microbial community composition of culture 1MN in the presence of different electron donor and acceptor combinations. The relative abundances of the MAG Dsb_1MN population and the *Thermoanaerobacteraceae* in the culture grown on 1-methylnaphthalene and ferrihydrite (top panel) were deduced from the average read coverage in the metagenome, which confirmed previous results obtained from fingerprinting by T-RFLP (14). The relative abundances in the absence of 1-methylnaphthalene were inferred from fingerprinting by T-RFLP and confirmed by amplicon sequencing (Fig. S6). **c** Fluorescence in situ hybridization (FISH) of the cable bacteria enrichment culture grown with elemental sulfur as electron donor and nitrate as electron acceptor stained with probe FliDSB194 specific for the MAG Dsb_1MN cable bacteria population. **d** Atomic force micrograph of filaments in culture 1MN grown with elemental sulfur and nitrate as electron acceptor showing the characteristic cell envelope of cable bacteria. The image displays the vertical deflection measured in contact mode

Cable bacteria are filamentous multicellular microorganisms belonging to the family *Desulfobulbaceae* [1]. They appear in redox gradients where the cells of one end of the filaments seemingly oxidize sulfide to sulfate [2]. The electrons from sulfide oxidation can be transported over several centimeters by long-distance electron transfer (LDET) to the sediment surface where they are used for oxygen or nitrate reduction [3–5]. The electrons are transported via conductive fibers in the periplasm leading to the distinctive morphology of a continuous ridge pattern over the whole length of cable bacteria [6]. Since their first discovery in sediments from Aarhus Bay [1], cable bacteria were found in many other marine sediments all over the world [7] but also in a freshwater stream in Denmark [8] as well as in groundwater contaminated with hydrocarbons [9]. So far, no attempts to cultivate cable bacteria in pure culture or in a stable enrichment culture have been successful. Based on genome sequencing, the cable bacteria known so far belong to a monophyletic sister clade of the genus *Desulfobulbus* with two proposed genera *Candidatus* Electrothrix and *Candidatus* Electronema [10, 11]. 16S rRNA gene sequences of groundwater cable bacteria formed a distinct phylogenetic clade with the closest cultivable relative *Desulfurivibrio alkaliphilus* [12]; a single-celled, rod-shaped alkaliphilic microorganism capable of sulfur disproportionation [12] and sulfide oxidation with nitrate as electron acceptor [13]. Surprisingly, we found 16S rRNA gene sequences of groundwater cable bacteria in the enrichment culture 1MN [14] that anaerobically degrades 1-methylnaphthalene or naphthalene with ferric iron as electron acceptor. This culture contains two dominant organisms affiliated to *Thermoanaerobacteraceae* and *Desulfobulbaceae* (Fig. 1b). The *Thermoanaerobacteraceae* were identified as the degraders of naphthalene by stable isotope probing experiments and the detection of putative genes encoding enzymes for naphthalene degradation [14]. The *Desulfobulbaceae* shared 16S rRNA gene identity of >98% with previously published sequences of groundwater cable bacteria (Fig. 1a) [9]. Since iron reduction and naphthalene oxidation are in stark contrast to the environmental conditions where cable bacteria are usually found, the discovery of groundwater cable bacteria in this chemo-organo-heterotrophic culture raised the question for their metabolic role. Our hypothesis was that sulfur disproportionation plays a major role in energy conservation of cable bacteria. Therefore, we enriched the cable bacteria in the absence of an organic electron source with elemental sulfur and Fe(OH)_3_ as sulfide scavenger or terminal electron acceptor. After four consecutive transfers, we performed substrate-turnover experiments with culture 1MN where we simulated the conditions that cells of the cable bacteria filaments might be facing along the geochemical gradients by adding sulfide, elemental sulfur, or thiosulfate as electron sources. In addition, we performed genome-resolved metagenomics of the enrichment culture 1MN and our cable bacteria enrichment culture and generated the first available, near complete genome (MAG Dsb_1MN) (Table S2, Fig. S3) of a groundwater cable bacterium, of which we elucidated the genetic potential.

## Materials and methods

### Cultivation of culture 1MN

The iron-reducing, 1-methylnaphtalene-degrading enrichment culture 1MN was enriched from a former coal gasification site in Gliwice, Poland [14]. It was grown in 125 ml serum bottles filled with 65 ml anoxic freshwater mineral medium [15] and sealed with butyl rubber stoppers under 80% N_2_ and 20% CO_2_ (Linde, Germany) atmosphere. The medium was reduced with 0.7 mM Na_2_S and buffered to pH 7 with 30 mM carbonate buffer. Twenty millimolars amorphous ferrihydrite [16] served as sole electron acceptor and 0.35 mM 1-methylnaphthalene was added as electron donor and carbon source. Fresh cultures were started by inoculation with 10% from a previous culture and incubated at 30 °C.

### Substrate-turnover experiments

To investigate if sulfur and iron cycles are coupled in culture 1MN and to elucidate the function of MAG Dsb_1MN, 2% (v/v) of a 1MN culture grown with ferrihydrite and 1-methylnaphthalene were transferred to fresh medium reduced with 0.7 mM Na_2_S and amended with elemental sulfur or thiosulfate in the presence or absence of ferrihydrite or nitrate as electron acceptor (Table 1). In the presence of Fe(OH)_3_, the reducing agent Na_2_S was abiotically oxidized to elemental sulfur or precipitated as black FeS. The media containing elemental sulfur were sterilized in an autoclave at 110 °C for 30 min to prevent melting of the sulfur. No organic substrate was provided for growth and all experiments of this study were conducted after four consecutive transfers to exclude carryover of the methylnaphthalenes. Each of the different cultivations was performed in two replicates inoculated with 2% (v/v) of the same source culture in order to start with the same microbial community composition and two abiotic controls.

**Table 1:** Incubation conditions for the substrate-turnover experiments with the cable bacteria enrichment culture

E-acceptor^a^	E-donor^a^	Reducing agent	pH
8 mM NO_3_−	3 mM S^0^	0.7 mM Na_2_S	6.4
8 mM NO_3_−	3 mM S_2_O_3_^2−^	0.7 mM Na_2_S	6.4
30 mM Fe(OH)_3_	3 mM S^0^	0.7 mM Na_2_S	6.4
30 mM Fe(OH)_3_	3 mM S_2_O_3_^2−^	0.7 mM Na_2_S	6.4
3 mM S^0^	10 mM FeCl_2_		8.0
8 mM S_2_O_3_^2−^	10 mM FeCl_2_		8.0

^a^In the absence of nitrate and Fe(OH)_3_, S^0^ and S_2_O_3_^2−^ served as both electron donors and electron acceptors

### Analysis of the products of the substrate-turnover experiments

Serum bottles were homogenized by manual shaking and 600 µl of the culture were sampled with a syringe through the stopper. Samples were processed immediately for further analyses to minimize oxygen exposure. For iron measurements, 20 µl sample were dissolved in 180 µl of 1M HCl for ~3 h. Fe(II) concentrations were determined with the ferrozine assay on a 96-well plate reader (Tecan, Switzerland) by measuring the absorbance at 560 nm [17, 18]. For sulfide analysis, 20 µl of sample were fixed in 400 µl of a 1% zinc acetate solution. Sulfide concentrations were measured within 2 h by the methylene blue method [19], which was downscaled to 96-well plate volumes [9]. To this end, 100 µl of the sample trapped in zinc acetate were mixed directly in the 96-well plate with 100 µl H_2_O, 25 µl 4-amino-N,N-dimethylaniline sulfate solution and oxidized to methylene blue with 25 µl of ferric ammonium sulfate solution. The absorbance of triplicate samples was measured at 670 nm on a 96-well plate reader. Sulfide concentrations were calculated from a standard curve derived from different dilutions of a 100 mM Na_2_S standard solution covering a range between 50 µM and 5 mM. However, only dissolved sulfide and easily soluble S^2−^ were measured by this method. For measuring total acid-volatile sulfides (AVS), 100 µl of sample were added to 7 ml of 6 M HCl in a tube with anoxic headspace containing a sulfide trap of 400 µl 10% (w/v) zinc acetate and incubated for 24 h. The trapped sulfide was quantified as described above by the methylene blue method using a FeS standard for calibration. For ion chromatography, 100 µl of sample were diluted in 900 µl MilliQ water in an Eppendorf tube, immediately put on ice, and centrifuged for 15 min at 12,000 rpm to remove iron particles and cells. Major anions (NO_3_^−^, NO_2_^−^, and SO_4_^2−^) and cations (NH_4_^+^) in the supernatant were measured by ion chromatography with a Dionex aquion system (Thermo Fisher Scientific, Dreieich, Germany).

### Atomic force microscopy

For atomic force microscopy, 2 ml of culture 1MN were fixed for at least 24 h in 2.5% glutaraldehyde at 4 °C and afterward centrifuged at 8000 rpm for 20 min. The supernatant was discarded and the pellet was resuspended in 200 µl MilliQ water. Twenty microliters of the cell suspension was dried for 2 h on a microscope glass slide and analyzed with an atomic force microscope (Nano Wizard, JPK Instruments, Germany) in contact mode using a CSC38/NO AL probe (Mikromasch, Tallinn, Estonia).

### DNA extraction, T-RFLP, and amplicon sequencing

For DNA extraction, at least 10 ml aliquots were centrifuged for 10 min at 18,000 × *g* at 4 °C. DNA was extracted from the pellet with a FastDNA Spin Kit for Soil (MP Biomedicals, Illkirch, France). 16S rRNA gene amplification and T-RFLP were performed as previously described using Ba27f (FAM-labeled) and 907r as primer for amplification and MSPI as restriction enzyme [20]. For amplicon sequencing, we used primers Pro341F and Pro805R [21] targeting 16S rRNA genes of prokaryotes. The first stage PCR was performed in KAPA HiFi Hot Start Ready Mix (Roche, Basel, Switzerland) by using 0.25 µM of each forward and reverse primers ligated to Illumina overhang adapters (Eurofins Genomics, Ebersberg, Germany) and 1 µl of extracted DNA as template in a total reaction volume of 25 µl. After an initial denaturation step at 94 °C for 5 min, the PCR was performed in 30 cycles of denaturation at 94 °C for 30 s, annealing at 55 °C for 30 s, and extension at 70 °C for 1 min, and a final extension at 70 °C for 5 min. The PCR amplicons were purified using MagSi-NGSPREP Plus magnetic beads (Steinbrenner, Wiesenbach, Germany) according to the Illumina 16S metagenomic sequencing library preparation guide (part no. 15044223 Rev. B) with the modification that the beads were resuspended in 42.5 µl of elution buffer EB (Qiagen, Hilden, Germany). Forty microliters of the supernatants were then taken for further analyses. The subsequent index PCR was performed using the Nextera XT DNA Library Preparation Kit v2 Set D (FC-131-2004) from Illumina (Munich, Germany) followed by a clean up according to the Illumina 16S metagenomic sequencing library preparation guide. DNA concentrations were measured with a Qubit fluorometer (Thermo Fisher Scientific, Dreieich, Germany). The samples were normalized to a concentration of 4 ng/µl and 5 µl of each sample were pooled in one ready-to-load sample, which was sequenced by GATC Biotech AG (Konstanz, Germany) on an Illumina Miseq platform. The demultiplexed raw reads of 250-bps length were processed using mothur by following the MySeq SOP [22, 23]. The quality-filtered and error-corrected sequences were clustered into operational taxonomic units (OTUs) at a defined cut-off level of 97% sequence similarity and classified by using the RDP classifier (mothur-formated trainset 16) [24]. Raw sequencing reads were deposited in the NCBI database in Bioproject ID PRJNA523091.

### Fluorescence in situ hybridization (FISH)

Cells were fixed in 2.5% final concentration of a 25% anoxic glutaraldehyde solution and stored at 4 °C for later analysis. For FISH, fixed samples were centrifuged at 8000 rpm for 30 min in an Eppendorf centrifuge, the supernatant was discarded, and the pellet was resuspended with MilliQ water in one fifth of the original volume. Twenty microliters of sample were transferred to wells of an eight-well microscope slide. The samples were dried at 46 °C for 2 h and dehydrated subsequently in 50, 70, and 98% ethanol for 3 min each. Hybridization and washing were done according to a previously published protocol [25] at a formamide concentration of 35%. We used different oligonucleotide probes for the detection of cable bacteria: probe DSB706 (Cy3, double labeled; Biomers, Ulm, Germany) for *Desulfobulbaceae* [26] in general and probe FliDSB194 (6-FAM, double labeled; Biomers) [9] for groundwater cable bacteria in particular. Both probes match 100% with the 16S rRNA gene sequence of the only OTU of *Desulfobulbaceae* present in the cable bacteria enrichments. Probe FliDSB194 was tested for its specificity in silico and is not expected to hybridize under the conditions used with *D. alkaliphilus* (two mismatches (MM)), *D. propionicus* (four MM), and *Ca*. Electrothrix (six MM) and no 16S rRNA gene sequence of any of these bacteria has been detected by amplicon sequencing. As negative control, we used probe NON338 [27] (6-FAM, double labeled; Biomers) representing the complementary sequence to EUB338 [28], the general probe for bacteria. As additional negative control, we also applied probe CFX1223 (6-FAM, double labeled; Biomers) [29] targeting the *Anaerolineaceae* from the phylum Chloroflexi, which were also present in the cable bacteria enrichment cultures at minor relative abundance. Both probes showed no hybridization with the cable bacterial filaments (Fig. S7). After the washing step, cells were counterstained with 2 µg µl^−1^ 4′,6-diamidin-2-phenylindol for 3 min and embedded in Citifluor AF1 (Citifluor, UK). Microscopy was performed with an eclipse epifluorescence microscope (Nikon, Melville, USA) using NiS elements software (version 4.10.01, Nikon) for imaging.

### Genome-resolved metagenomics

We performed genome-resolved metagenomics on DNA extracted during a previously published SIP experiment of culture 1MN grown on naphthalene for 72 days [14]. Library preparation and 150-bps paired-end Illumina HiSeq sequencing were performed at GATC (Konstanz, Germany). Raw reads were trimmed and quality filtered with bbduk (http://jgi.doe.gov/data-and-tools/bbtools/) and SICKLE version 1.21 (https://github.com/najoshi/sickle), and assembled and scaffolded with metaSPADES version 3.10.1 at default settings [30]. For scaffolds longer than 1 kb, 16S rRNA genes were identified using CMsearch [31] and gene prediction was performed with prodigal in the meta mode (-p meta) [32]. The predicted genes were taxonomically and functionally annotated using diamond blastp [33] against the Uniref100 database [34]. The scaffolds were binned into draft bins using a tetranucleotide-frequency based emerging self-organizing map [35] and further curated using GC, taxonomy, and coverage information. The resulting bins were curated for scaffolding errors using ra2 [36] and again curated using GC, taxonomy, and coverage information. Quality of genomes was evaluated using 51 bacterial [37] and 38 archaeal single copy genes [38].

### Resequencing and strain analysis

We used the 1MN culture as inoculum for enrichment cultures predicted to select for cable bacteria physiology. To confirm the target cable bacteria population (MAG Dsb_1MN) was present in the new enrichment cultures (lacking an organic carbon source) we sequenced the metagenomic DNA followed by read QC as described above. Using GC content, coverage, and taxonomy information, we also reconstructed a near complete genome of this dataset. To test the similarity between the genome enriched with sulfur and the one of the organism that was originally found in the 1MN culture we calculated the average nucleotide identity (http://enve-omics.ce.gatech.edu/ani/) between the two reconstructed genomes (window size 1000 bs, step size 200 bps, minimum length 700 bps, minimum identity 70%, minimum alignments 50). In addition, we visualized the similarity between the two genomes using circoletto [39], based on blastn (*e*-value cutoff 1-e−10). Then, we used stringent read mapping [40] and filtering for a maximum of three mismatches per read (equivalent to a sequencing error rate of 2%). Newly generated reads were aligned to the reconstructed cable bacteria genome MAG Dsb_1MN. SNP, insertion and deletion were calculated using default settings in the Geneious software [41].

### Availability of metagenomic data

Draft genome sequences were deposited in the NCBI database in Bioproject ID PRJNA475330 with the biosample accession numbers SAMN10188309, SAMN10188310, SAMN10188311, and SAMN10188512. The cable bacterial genome was uploaded to the Genoscope platform MAGE [42, 43] and annotated. Metabolic pathways were predicted by KEGG [44] pathway profiling of MAGE annotations.

### Phylogenetic analyses

Phylogenetic trees of 16S rRNA gene sequences were calculated in the MEGA X software [45] using the maximum likelihood method based on the Tamura-Nei model [46]. For phylogenetic placement of cable bacteria on the tree of life (beyond 16S rRNA gene analyses), we extracted 16 ribosomal proteins [47] using established methods [37]. The ribosomal proteins were aligned [48] with reference sequences of an in-house database consisting of 3800 dereplicated public genomes from previous publications [38, 47, 49]. Alignments were end-trimmed and manually inspected before concatenating them and building a tree using FastTree version 2.1.8 [50]. The resulting two-domain tree was pruned to a monophyletic subclade reflecting the position of the cable bacteria.

## Results

After only four consecutive transfers of enrichment culture 1MN with sulfur as electron source, one of the original four OTUs was no longer detectable (*Thermoanaerobacteraceae*) and our target of putative cable bacteria was enriched to a relative amplicon abundance of >90% (Fig. 1a, b, Fig. S6). Complementary FISH with probe FliDSB194 [9] specific for the cable bacterium and probe DSB706 [26] specific for the family *Desulfobulbaceae* (both matched 100% with the 16S rRNA gene sequence of MAG Dsb_1MN) demonstrated that the cable bacterium was present in several hundred micrometer long filaments, but also in shorter filaments as well as in individual cells (Fig. 1c, Figs. S5, S7, and S8). The relative proportion of these cell forms in the cable bacteria enrichment culture changed over the course of the experiment (Fig. S7). The lengths of the filaments as well as the proportion of filaments over single cells seemed to increase with incubation time. All observed filaments were positive for probes FliDSB194 and DSB706 indicating that all cell forms belonged to the same cable bacteria represented by the genome sequence MAG Dsb_1MN (see below). Neither the filaments nor the single cells hybridized with probe NON338 as a negative control (Fig. S7). Atomic force microscopy revealed that all observed filaments showed the typical cell envelope with the continuous ridge pattern [1] confirming their morphology as cable bacteria (Fig. 1d). So far, we were not able to detect the ridge patterns for single cells of the cable bacteria.

**Fig. 2:**
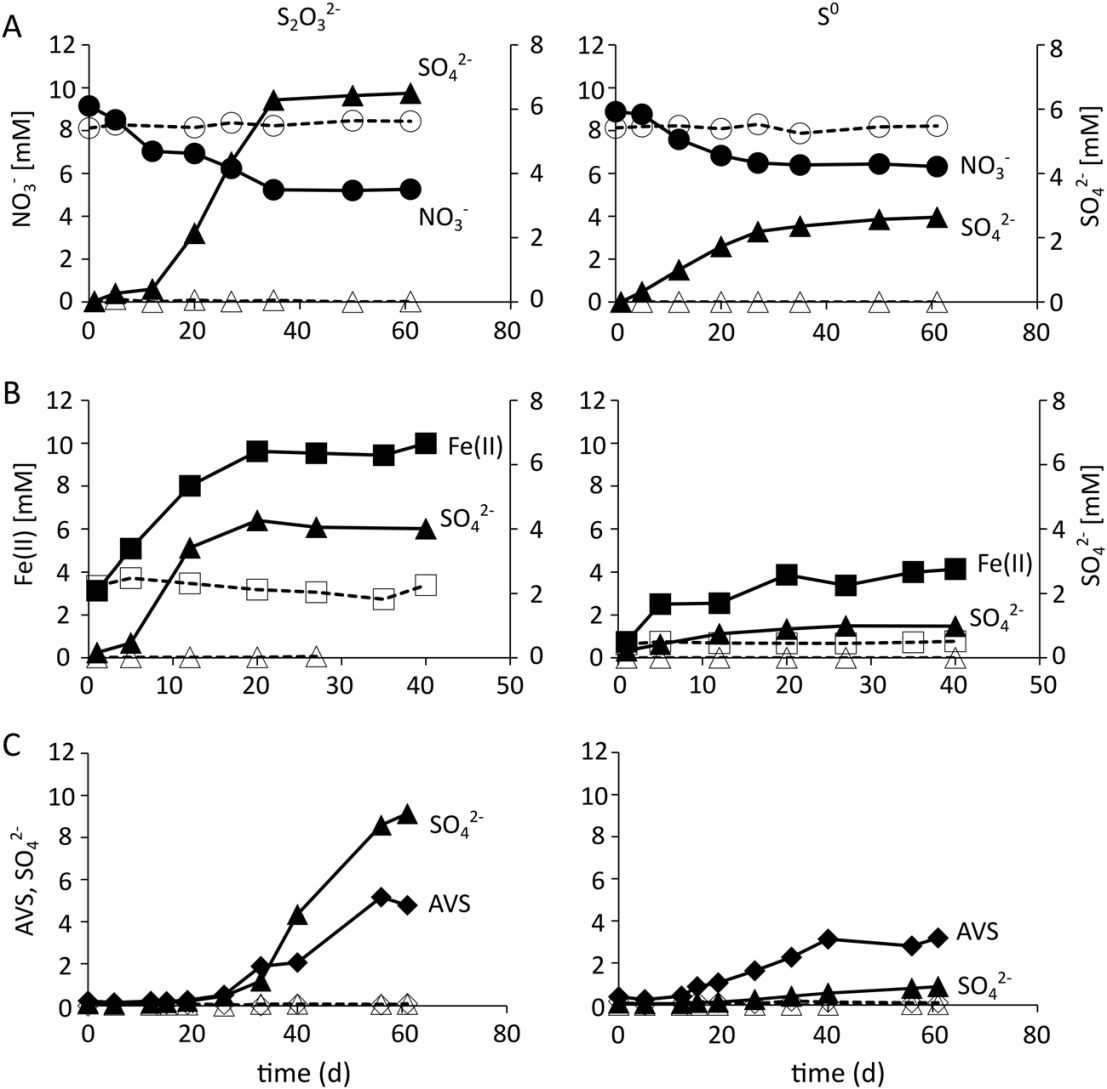
Development of concentrations of electron acceptor and sulfur species in the 1MN culture. Active cultures (filled symbols, solid lines) supplied with thiosulfate (left panel) or elemental sulfur (right panel) and electron acceptors **a** nitrate, **b** ferrihydrite, or **c** at disproportionation conditions in comparison to abiotic controls (open symbols, dashed lines). Concentrations of nitrate (circles), Fe(II) (squares), sulfate (triangles), and acid-volatile sulfides (AVS, diamonds) of one representative incubation is shown over the course of the experiment. Graphs of other replicate incubations are shown in Fig. S1

**Fig. 3:**
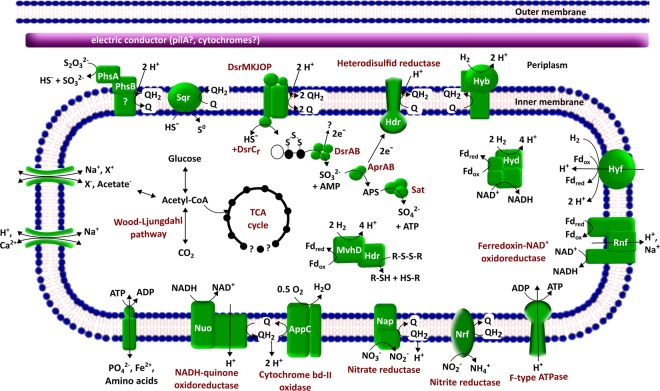
Metabolic potential of groundwater cable bacteria (MAG Dsb_1MN). The pathways were inferred from KEGG [44] pathway profiling on the Genoscopes platform MAGE [42, 43]. The functions of enzymes involved in sulfur metabolism were inferred from the literature [13, 67]

When the cable bacteria enrichment culture was provided with elemental sulfur or thiosulfate as electron source and nitrate as electron acceptor, the culture showed production of sulfate with concomitant reduction of nitrate to ammonia (Fig. 2a, Fig. S1A, Table S1). However, the ammonium recovery was only 30–60% of the nitrate reduced (Table S1), which might have been caused by degassing of ammonia from the medium. Since nitrite was only detected at low concentrations (<100 µM), incomplete nitrate reduction to nitrite cannot explain this observation. Nevertheless, the decrease in nitrate fits to the stoichiometric oxidation of sulfur by dissimilatory nitrate reduction to ammonium (DNRA) (Fig. 2a, Fig. S1A, Table S1). This overall reaction might imply that the cable bacteria simply perform sulfur oxidation with nitrate as electron acceptor. Indeed, the genome of the cable bacteria (MAG Dsb_1MN) encodes for dissimilatory nitrate reduction to ammonia and a complete sulfate reduction pathway, which could have been operated in reverse (Fig. 3, Table S3). During days 5–12 of the incubation, one of the two replicate incubations with S_2_O_3_^2−^ as electron donor showed a high drop in NO_3_^−^ concentration compared with a relatively low increase in SO_4_^2−^ concentrations. This was only observed once and might have been caused by issues during analysis of SO_4_^2−^ on day 12. When we added ferric iron instead of nitrate as electron acceptor to cultures with sulfur or thiosulfate as substrate for sulfur disproportionation, again an oxidation of elemental sulfur to sulfate occurred coupled to the reduction of ferric iron. However, the stoichiometry and the production of AVS indicated an abiotic reduction of ferric iron with sulfide produced during disproportionation (Fig. 2b, Fig. S1B, Table S1). Indeed, we observed sulfur and thiosulfate disproportionation when free sulfide was kept very low by Fe(II) as a scavenger, indicated by a simultaneous increase of sulfate and AVS in a 1:3 or 1:1 ratio, respectively (Fig. 2c, Fig. S1C, Table S1). However, with longer incubation time less sulfide was measured than expected (Table S1), which might have been caused by: (a) degassing of sulfide, (b) electrons used for autotrophic carbon fixation, or (c) pyrite formation, which has been shown previously [51].

Sulfide can be toxic to cells but also thermodynamically inhibiting sulfur disproportionation. To test the effect of different sulfide concentrations on cable bacteria performing sulfide oxidation with nitrate as electron acceptor (as previously described for the closest cultivated relative *Desulfurivibrio alkaliphilus* [13]), we incubated our cable bacteria enrichment culture with nitrate as electron acceptor, elemental sulfur, or thiosulfate as electron donors, and different concentrations of sulfide from 0 to 2 mM as potentially inhibiting sulfide background concentration. Sulfur oxidation or sulfur disproportionation did only take place at dissolved sulfide concentrations lower than 120 µM and was completely inhibited at concentrations higher than 300 µM at pH 6.4 (Fig. S2).

To further elucidate the physiology of the cable bacteria (genome MAG Dsb_1MN), we investigated the metagenome of DNA extracted during the growth phase of culture 1MN on naphthalene after 72 days of incubation. We received 6.7 million paired reads, which assembled into 141 scaffolds longer than 1 kbps. Binning of the scaffolds based on tetranucleotide frequencies led to four clearly separated genome bins, each belonging to a different phylum (Fig. S3). The genome MAG Dsb_1MN revealed hits for 2740 protein-coding genes against the Uniref100 database. Thirty-seven percent of these genes were coded for uncharacterized proteins. With an estimated genome completeness of >98%, MAG Dsb_1MN shows the so far highest completeness of all cable bacterial genomes [11]. We additionally sequenced the metagenome of our cable bacteria enrichment culture after substrate-turnover experiments and reconstructed a genome that has a 99.9% average nucleotide identity (0.08% SD, two way) with the original genome. Whole genome alignments based on blast are provided in Fig. S9 and indicated that the recovered genomes are nearly identical (only a few SNPs were detected, Fig. S4). Within the community of the cable bacteria enrichment, we detected four different organisms based on ribosomal protein S3 (rpS3) markers. The cable bacterium was the dominant organism with a coverage of 447 for the rpS3-carrying scaffold (on average). A Chloroflexi sequence showed a coverage of 94 and the two other sequences (Actinobacteria and Verrucomicrobia) were both detected with 11-fold coverage per rpS3 scaffold. Hence, also the metagenomic data indicated that the cable bacteria (MAG Dsb_1MN) from culture 1MN were successfully enriched with sulfur as electron source.

Metabolic pathway prediction confirmed that the cable bacteria (genome MAG Dsb_1MN) have potential for versatile sulfur metabolism including all proteins of dissimilatory sulfate reduction, a sulfide-quinone reductase (SQR) and a thiosulfate reductase (PHS) (Fig. 3, Table S3). The genome codes for all proteins of DNRA confirming the results from our substrate-turnover experiments, which showed ammonium production with nitrate as electron acceptor (Tables S1 and S3). Genes for a terminal cytochrome bd-II oxidase indicate the potential of oxygen reduction (Fig. 3, Table S3). Intriguingly, the genome contains genes for four different types of hydrogenases suggesting hydrogen as alternative electron donor for cable bacteria. Specifically, electron bifurcating F420-non-reducing [52] and Hyd-type hydrogenases [53] in the cytoplasm might provide reduced ferredoxin and NADH for autotrophic CO_2_ fixation (Fig. 3, Table S3). Membrane bound Hyb- and Hyf-type hydrogenases could couple hydrogen oxidation to quinone, NAD^+^, or ferredoxin reduction. Alternatively, the enzymes could produce hydrogen when operated in reverse. In addition, a ferredoxin-NAD^+^ oxidoreductase (Rnf) complex could couple ferredoxin oxidation and NAD^+^ reduction to energy conservation by dislocation of protons or sodium ions [54]. The proton motive force could be exploited for ATP generation by an F-type ATPase (Fig. 3, Table S3) [55].

Genes for the complete pathways of glycolysis/gluconeogenesis were present in the genome (Tables S1 and S3). The genome is lacking a complete TCA-cycle since we could not detect genes for fumarate reductase and succinate synthase (Fig. 3). Genome MAG Dsb_1MN shows potential for CO_2_ fixation reflected by the presence of all genes of a Wood–Ljungdahl pathway. So far, the composition of the conductive structures and the respective genes for a LDET are unknown. However, c-type cytochromes have been suggested to be involved in electron conduction and as a capacitor [5]. We found 15 genes coding for different c-type cytochromes of which, for instance, multiheme cytochromes DmsE and PpcG are known to be involved in periplasmatic electron transfer during iron reduction (Table S3) [56, 57]. The genome encodes also for PilA, which might be involved in extracellular electron transport (Table S3) [58].

## Discussion

In laboratory enrichment cultures as well as in contaminated aquifers, hydrocarbon-degrading organisms are frequently associated with highly abundant bacteria of the family *Desulfobulbaceae* closely related to groundwater cable bacteria [9, 14, 59]. We enriched groundwater cable bacteria originating from the iron-reducing, naphthalene-degrading culture 1MN to more than 90% in relative abundance, only with elemental sulfur as electron source and ferrihydrite as electron acceptor and sulfide scavenger. This supports the recent proposal that the *Desulfobulbaceae* might be involved in sulfur cycling during 1MN degradation in culture 1MN [14].

Specific FISH for groundwater cable bacteria revealed that the *Desulfobulbaceae* were present as several hundred µm long filaments, but also shorter filaments and single cells. This contrasts with findings for marine cable bacteria where to our knowledge no single-celled state was observed so far. Atomic force microscopy revealed the typical cable bacterial morphology with the continuous ridge pattern for our cable bacteria enrichment, similar to the originally discovered monophyletic cluster of cable bacteria 16S rRNA sequences from marine and freshwater [10].

Since the discovery of cable bacteria, it has been a major question how the cells in the middle of the filaments conserve energy because there is no visible reaction taking place in the suboxic zone of the geochemical gradient. Obvious reactions are only the sulfide oxidation at the anodic end and oxygen reduction at the cathodic end of the filaments. In our substrate-turnover experiments with the cable bacteria enrichment culture we simulated the conditions that cells in the cable bacteria filament are facing along the geochemical gradients. The results presented here provide clear evidence that cable bacteria can conserve energy by sulfur or thiosulfate disproportionation with FeCl_2_ as sulfide scavenger (Fig. 2, Fig. S1). In this case, energy could be conserved in all cells via substrate-level phosphorylation in the last step of a reverse sulfate reduction pathway, when adenosinephosphosulfate is converted to sulfate and ATP by a reverse operating sulfate adenylyltransferase (Fig. 3, Table S3). We thus propose that the cable bacterial cells oxidize sulfide to elemental sulfur in a first step that is coupled by LDET to oxygen reduction or nitrate reduction to ammonium. The sulfur is then disproportionated by a reverse sulfate reduction pathway producing sulfate and sulfide. Hence, the role of LDET might be to provide elemental sulfur for the energy-conserving sulfur disproportionation. LDET thus mainly serves as an electron sink or acceptor for sulfide oxidation by cable bacteria but no energy can be conserved in this step. A similar mechanism has been demonstrated recently for *Desulfurivibrio alkaliphilus* [13]. Transcriptomics indicated that *D. alkaliphilus* oxidizes sulfide to elemental sulfur in a first step, which can then be either disproportionated or oxidized with nitrate as electron acceptor [13].

In contrast to *D. alkaliphilus*, our cable bacteria enrichment culture showed no sulfur disproportionation or oxidation of sulfide at concentrations higher than 300 µM indicating a thermodynamic or toxic inhibition of sulfur disproportionation by free hydrogen sulfide. Since this inhibition is complete and inhibiting energy conservation, the cable bacteria can also not slowly oxidize the sulfide to lower concentrations where it could start off with growth. At the slightly acidic pH of 6.4 during our substrate-turnover experiments with nitrate most of the sulfide was present as gaseous H_2_S, which can pass cell membranes [60] and consequently inhibit sulfur disproportionation. In contrast, at the alkaline pH during cultivation of *D. alkaliphilus* (>pH 9.5) almost all sulfide is present as HS^−^ or S^2−^, which cannot pass the cell membranes. This might be the reason why *D. alkaliphilus* can grow at higher sulfide concentrations, whereas our cable bacteria cannot [13, 61].

Recently, three genomes of marine *Ca*. Electrothrix and one genome of *Ca*. Electronema have been published based on single-cell sequencing and metagenomics [11]. In the following, we provide an overview of the similarities and differences of these genomes to the genome MAG DSB_1MN of our cable bacteria. While the genome size of 3.1 Mbps of MAG DSB_1MN is within the range of 2.7–4.0 Mbps reported for other cable bacteria, MAG DSB_1MN has a clearly higher GC content of 57% compared with ~50% already distinguishing MAG DSB_1MN from other cable bacteria. MAG DSB_1MN has several genes which might have been lost, reduced, or replaced in other cable bacteria such as the glycolytic enzyme enolase, a complete DsrKMJOP complex, and the NADH-quinone oxidoreductase (Nuo) enzyme complex (Table S3) [11]. Like in other cable bacteria and in *D. alkaliphilus*, an SQR might oxidize sulfide to elemental sulfur and the sulfate reduction pathway might be operated in reverse for energy conservation. No reverse-type dissimilatory sulfite reductase was observed, which is in accordance to other cable bacteria, *D. alkaliphilus*, and also other sulfur disproportionating *Desulfobulbaceae* such as *D. propionicus*. Kieldsen et al. [11] suggested energy conservation by sulfur disproportionation by a polysulfide reductase when cable bacteria are disconnected from electron acceptors. So far, we were not able to detect genes encoding for this enzyme in MAG DSB_1MN. One of the main questions since the discovery of cable bacteria is about the composition of the electron conductor. Based on metagenomic and proteomic data, Kieldsen et al. hypothesized electrically conductive type IV pili (e-pili) might form conductive superstructures in the periplasm. Our genomic data of MAG DSB_1MN also allow for this possibility, since we also found the gene coding for PilA in the genome. The amino acid sequence shows the same distribution of aromatic amino acids like electrically conductive e-pili (Fig. S10) [58].

Interestingly, genome analysis revealed genes for hydrogenases indicating the potential of MAG DSB_1MN to use hydrogen as alternative electron donor (Fig. 3, Table S3). Hydrogen might be an alternative electron source for cable bacteria in organic-rich habitats dominated by fermentation. However, this is in contrast to the genomes of marine and freshwater cable bacteria where a cytoplasmatic hydrogenase was detected only in *Ca. E. aarhusiensis* and periplasmatic hydrogenases were absent [11]. The presence of a complete Wood–Ljungdahl pathway for CO_2_ fixation, which is in accordance to previously published genomes [11], and the absence of an organic C-source in our enrichment culture strongly indicates the capability of MAG DSB_1MN of a chemo-litho-autotrophic metabolism.

Our cable bacteria enrichment culture was also capable of nitrate reduction to ammonium, which was confirmed by genes encoding for nitrate and nitrite reductases in genome MAG DSB_1MN. Although we did not test for oxygen as electron acceptor, genes encoding for cytochrome bd oxidase indicate that these organisms can reduce oxygen as terminal electron acceptor (Fig. 3, Table S3). Nevertheless, groundwater cable bacteria showed oxygen reduction in laboratory incubations of aquifer sediments [9]. Intriguingly, genes encoding for a cytochrome bd oxidase for oxygen as electron acceptor were absent in the genomes of *Ca*. Electrothrix and *Ca*. Electronema [11].

**Fig. 4:**
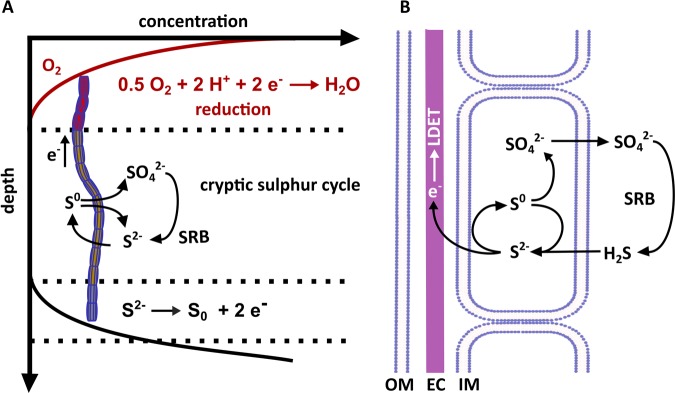
Conceptual model for energy conservation in groundwater cable bacteria. **a**, **b** Filaments span the suboxic zone by a long-distance electron transfer. Within the suboxic zone and the anodic zone, sulfide is oxidized to elemental sulfur, which is then used to conserve energy by sulfur disproportionation via a reverse sulfate reduction pathway. At the cathodic end, oxygen or nitrate reduction take place as electron accepting process for the LDET. Sulfide is provided all along the filament by sulfate-reducing bacteria. The sulfate is recycled by the cable bacteria providing a cryptic sulfur cycle in the suboxic zone. IM inner membrane; OM outer membrane; EC electric conductor

These results allow us to suggest a new model for energy conservation of cable bacteria, which provides an explanation of how each cell within the cable bacterial filament can conserve energy (Fig. 4). Near the surface, cable bacteria perform the cathodic reaction, i.e., the reduction of oxygen and nitrate to water and ammonium, respectively. So far, it is unclear if cable bacteria conserve energy from oxygen reduction. For instance, closely related species such as *Desulfobulbus propionicus* can reduce oxygen but show no growth with oxygen as electron acceptor [62]. We propose that below the cathodic zone elemental sulfur is disproportionated to sulfate and sulfide, whereas the sulfide is again oxidized to sulfur by LDET. The elemental sulfur can either be produced abiotically by fluctuating redox conditions or by a LDET by the cable bacteria themselves. Hence, the apparent overall reaction at the anodic part of the filaments is a net oxidation of sulfide to sulfate but energy is most likely conserved by sulfur disproportionation only. In natural sediments, chemo-organo-heterotrophic, sulfate-reducing bacteria will be abundant all along the cable bacteria filament and oxidize organic material with concomitant reduction of sulfate to sulfide (Fig. 4) [63]. We propose that all cells of the cable bacteria can oxidize this sulfide to elemental sulfur by LDET and the electrons are channeled through the cable filaments to the oxygen- or nitrate-reducing cathodic end. In fact, this pathway provides an explanation for energy conservation throughout the entire filament.

The energy-conserving sulfur disproportionation reaction requires low sulfide concentrations [64]. This suggests that in sediments the anodic oxidation of sulfide is limited to the suboxic zone and a narrow zone at the measurable end of the sulfide gradient (Fig. 4), which is characterized by low concentrations but high fluxes of sulfide. Hence, the functioning of cable bacteria relies on a delicate equilibrium between the rate of electron removal by LDET (and consequent oxygen or nitrate reduction rates) and the sulfide reduction rates by sulfate reducers (Fig. 4). Either a decrease of LDET, by, e.g., lower oxygen supply, or higher sulfate reduction rates could lead to increased sulfide concentrations along the filaments and immediate inactivation of the cable bacteria function. This might explain the frequently observed sudden disappearance of cable bacteria populations and LDET in marine sediments [65, 66].

## Supplementary information

Supplementary results

Phylogenetic tree
