# Supplementary material for: Groundwater cable bacteria conserve energy by sulfur disproportionation
Source: ISME J. 2019 Nov 14;14(2):623–34. doi: 10.1038/s41396-019-0554-1 (PMC6976610; doi:10.1038/s41396-019-0554-1)
Supplement: Supplementary file 1 — Supplementary results [file 41396_2019_554_MOESM1_ESM.docx]

**Groundwater cable bacteria conserve energy by sulfur disproportionation**

Hubert Müller^1^, Sviatlana Marozava^2^, Alexander J. Probst^1^, and Rainer U. Meckenstock^1^*

^1^University of Duisburg-Essen, Biofilm Center, Universitätsstr. 5, 45141 Essen, Germany

^2^Institute of Groundwater Ecology, Helmholtz Zentrum München, Ingolstädter Landstraße 1, 85764 Neuherberg, Germany* Corresponding author: University of Duisburg-Essen, Biofilm Center, Universitätsstr. 5, 45145 Essen, Germany. Tel: +49 (201) 183-6601; Fax: +49 (201) 183-6603; E-mail: [rainer.meckenstock@uni-due.de](mailto:rainer.meckenstock@uni-due.de)

The authors declare no conflict of interest.

**Supplementary results**


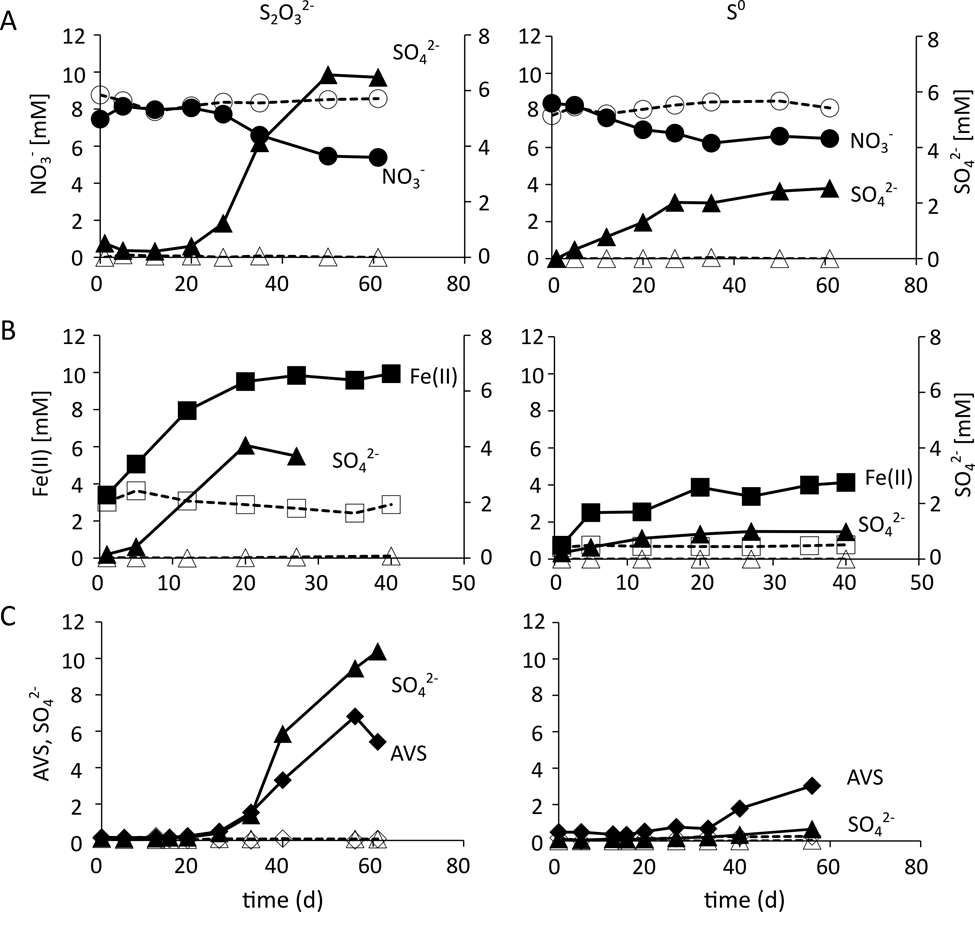


**Fig. S1** Development of concentrations of electron acceptor and sulfur species in the cable bacteria enrichment culture. Active cultures (filled symbols, solid lines) supplied with thiosulfate (left panel) or elemental sulfur (right panel) and electron acceptors **A**) nitrate, **B**) ferrihydrite, or **C**) at disproportionating conditions in comparison to abiotic controls (open symbols, dashed lines). Concentrations of nitrate (circles), Fe(II) (squares), sulfate (triangles), and acid volatile sulfides (AVS, diamonds) of one representative incubation is shown over the course of the experiment. Graphs of replicate incubation of Fig. 2 in the main text.

Table S1. Changes in concentrations of redox active compounds during different redox experiments performed with the cable bacteria enrichment culture

| **e-acceptor,**  **e^-^-donor** | **Initial HS^-^ [mM]** | **Fe(II) [mM]** | **NO_3_^-^ [mM]** | **NH_4_^+^ [mM]** | **SO_4_^2-^ [mM]** | | **AVS [mM]** | **e- recovery** |
| --- | --- | --- | --- | --- | --- | --- | --- | --- |
| NO_3_^-^, S_0_ | 0.1 | n.a. | -2.2±0.5 | 0.7±0.1 | 2.6±0.1 | | n.a. | 89% |
| NO_3_^-^, S_2_O_3_^2-^ | 0.1 | n.a. | -3.0±1.3 | 1.9±0.1 | 6.5±0.0 | | n.a. | 108% |
| NO_3_^-^, S_0_ | 0.4 | No growth | | | | | | |
| NO_3_^-^, S_2_O_3_^2-^ | 0.3 | No growth | | | | | | |
| Fe(OH)_3_, S_0_ | 0.0 | 2.8±0.9 | n.a. | n.a. | 0.7±0.1 | | 0.6±0.4 | 105% |
| Fe(OH)_3_, S_2_O_3_^2-^ | 0.0 | 6.7±0.2 | n.a. | n.a. | 3.9±0.3 | | 2.9±0.8 | 85% |
| S_0_ (FeCl_2_)^*^ | 0.0 | n.a. | n.a. | n.a. | 0.7±0.3 | | 2.0±1.1 | 105% |
| S_0_ | 1.1 | No growth | | | | | | |
| S_2_O_3_^2-^ (FeCl_2_)^*^ | 0.0 | n.a. | n.a. | n.a. | | 9.6±0.9 | 4.9±0.5 | 196% |
| S_2_O_3_^2-^ | 1.1 | No growth | | | | | | |

n.a.: not applied; initial HS: concentration of dissolved sulfide at the time of inoculation, AVS: acid-volatile sulfides; ^*^10 mM FeCl_2_ was used as sulfide scavenger and reducing agent instead of Na_2_S


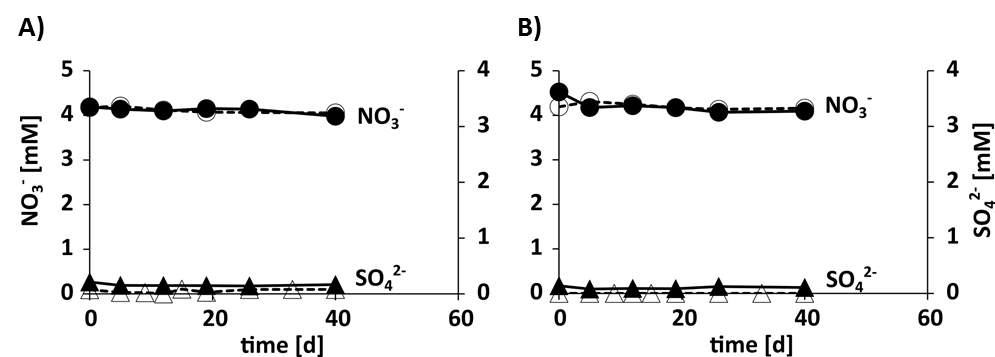


**Fig. S2** Inhibition of growth of the cable bacteria enrichment culture in the presence of 300 µM of dissolved sulfide with nitrate as electron acceptor and elemental sulfur **A)** and thiosulfate **B)** as electron donor. This was indicated by constant nitrate (circles) and sulfate (triangles) concentrations in active incubations (filled symbols) as well as in abiotic controls (open symbols). Data points depict means of two biological replicates. Standard deviations were smaller than the size of the symbols.

**Table S2. Results from binning of the assembled 1MN metagenome**

|  | ***Thermoanaero-bacteraceae*** | ***Desulfobulbaceae (MAG Dsb_1MN)*** | ***Atopo-biaceae*** | ***Anaero-lineaceae*** |
| --- | --- | --- | --- | --- |
| **Length** | 3.1 Mbp | 3.1 Mbp | 2.2 Mbp | 3.3 Mbp |
| **Number of scaffolds*** | 54 | 24 | 7 | 14 |
| **N50** | 0.1 Mbp | 0.2 Mbp | 0.6 Mbp | 1.0 Mbp |
| **Coverage** | 214 | 314 | 13 | 20 |
| **Estimated completeness** | <100% | 98% | <100% | <100% |
| **Genes (Uniref100)** | 2,743 | 2,740 | 2,012 | 2,879 |
| **Uncharacterized proteins** | 30% | 37% | 26% | 57% |

***** > 1000 base pairs


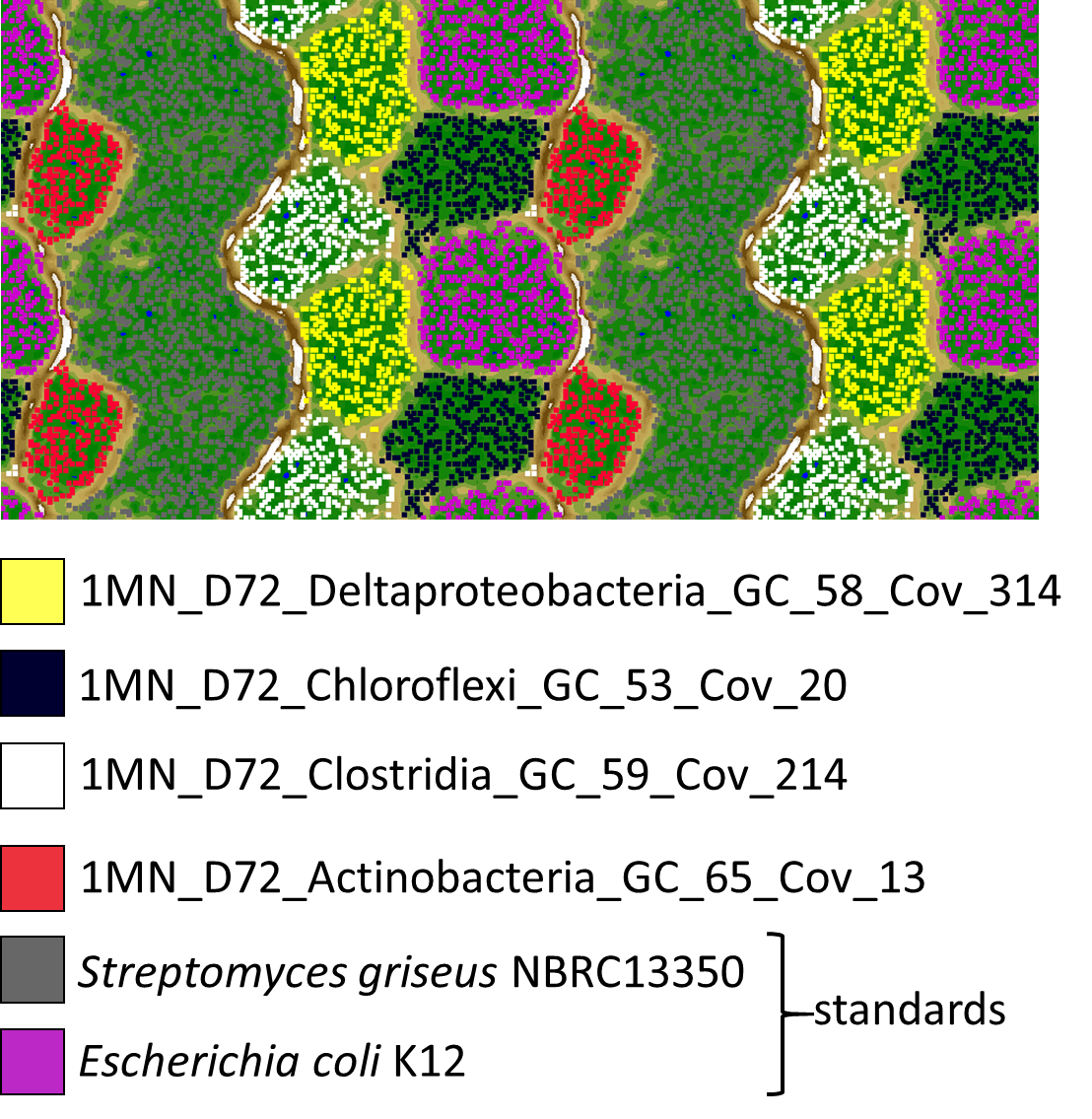


**Fig. S3** Confirmation of the binning approach using a tetra-frequency-based Emergent Self-Organizing Map (ESOM)(1). The coloring is according to the legend, *E. coli* and *S. griseus* were used as standards. 1MN_D72_Deltaproteobacteria_GC_58_Cov_314 is the genome of the cable bacterium MAG Dsb_1MN.

**
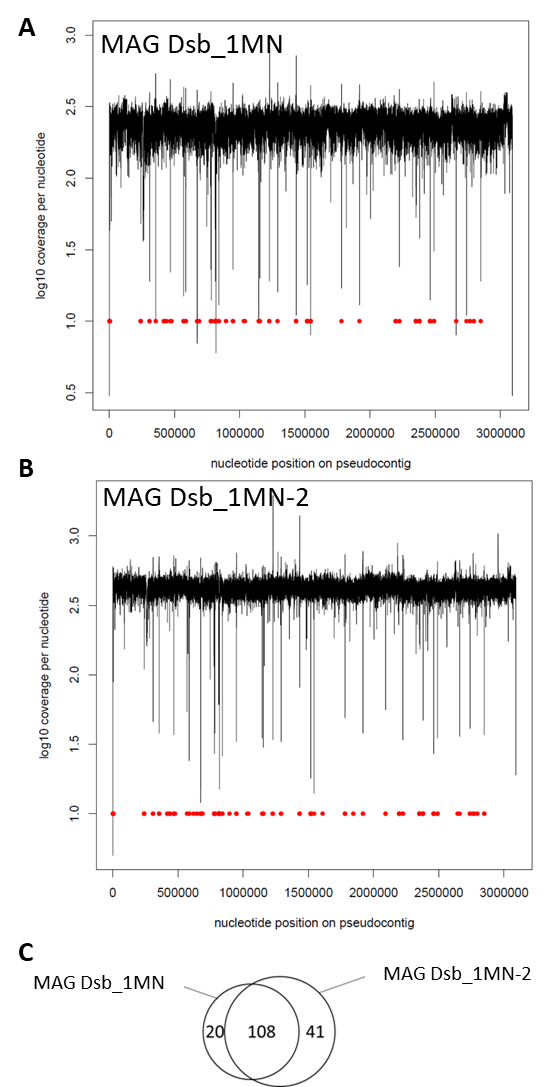
**

**Fig. S4** Variations of the population genome MAG Dsb_1MN (cable bacterium) under different cultivation conditions. **A), B)** Coverage of the pseudocontig (concatenated scaffolds with 100 Ns in-between) of the respective culturing conditions. Red points indicate positions with SNPs, insertions or deletions. After enrichment of the cable bacteria with nitrate as electron acceptor, the coverage increased along with several SNPs. However, the full genome was covered indicating that MAG Dsb_1MN was selectively enriched during the enrichment process. **C)** Number of unique SNPs, insertions, and deletions including their respective positions. 108 of the SNPs, insertions, and deletions were shared between the cultures being indicative that the enrichment process was successful. [Calling of SNPs, insertions, and deletions was performed in Geneious (2) with default settings.]


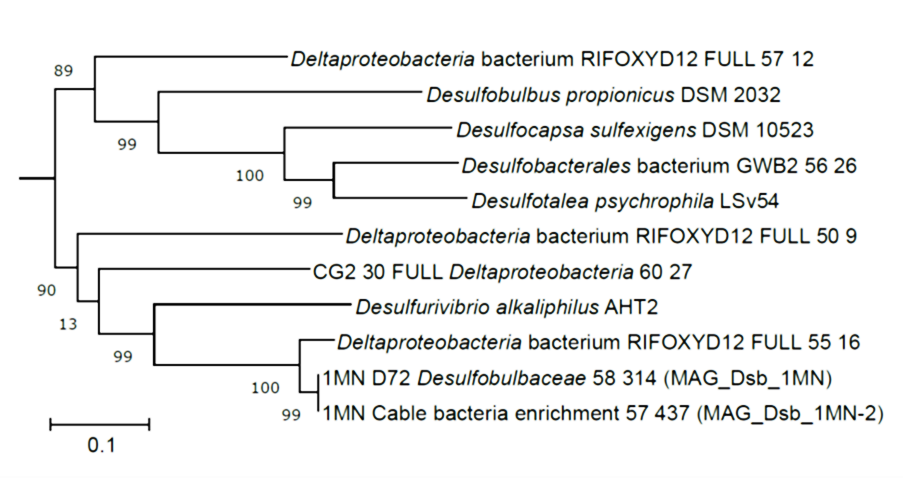


**Fig. S5** Phylogenetic placement of cable bacteria genomes based on 16 concatenated ribosomal proteins (for details please see methods). The two recovered genomes of the initial *Desulfobulbaceae* from culture 1MN and the enriched cable bacteria share the same phylogenetic position. Scale bar represents the number of substitutions per site. A full tree is also provided in the supplementary information.


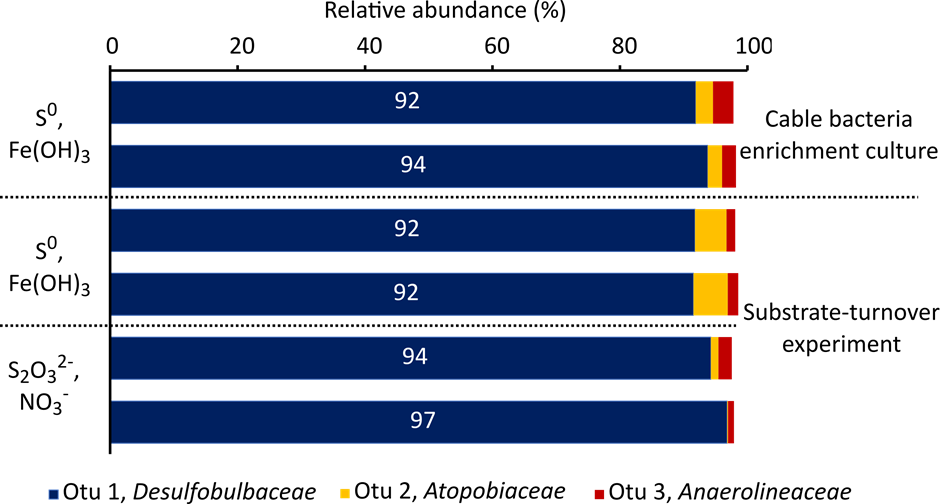


**Fig. S6** Purity of the cable bacteria enrichment culture determined by 16S rRNA gene sequencing before and after the substrate turnover experiment. OTU 1 representing cable bacteria dominated all cultures with more than 90% in relative abundance.


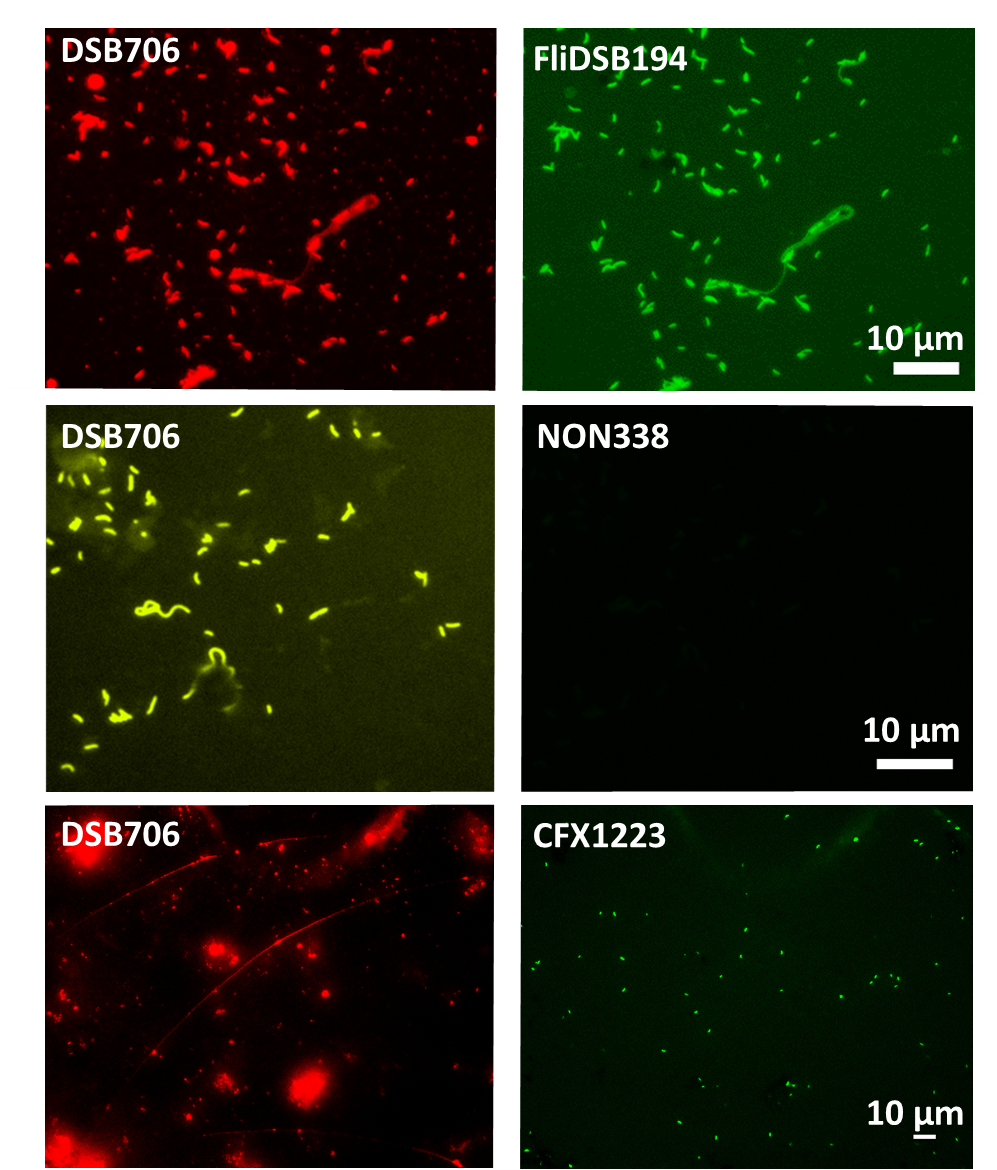


**Fig S7.** Test of specificity of the probes used for FISH on the same cable bacteria enrichment culture which was also used for AFM and FISH shown in culture. Pictures in each line show the same region of interest take with different filters. Cable bacterial filaments as well as single cells hybridized with both, FliDSB194 (6-Fam, green fluorescence) and DSB706 (Cy3, yellow-orange fluorescence) for groundwater cable bacteria and *Desulfobulbaceae* respectively. In comparison, probe NON338 (6-FAM) as a control was negative. Probe CFX1223 (6-Fam) targeting the *Anaerolineaceae* in the culture hybridized with single cells but not with the filaments. Please note that the different colours of the DSB706 images resulted from the two different filters Cy3 and DsRed used for image acquisition in order to exclude false positive signals by overlapping emmisions of the fluorophores. DsRed has a slightly higher emmision wavelength of 583 nm compared to 570 nm of Cy3 and has lower overlap to the 6-Fam emission. Yellow indicates the Cy3 filter at 570 nm whereas red images were taken by the DS We used a formamide concentration of 35% in the hybridization buffer.


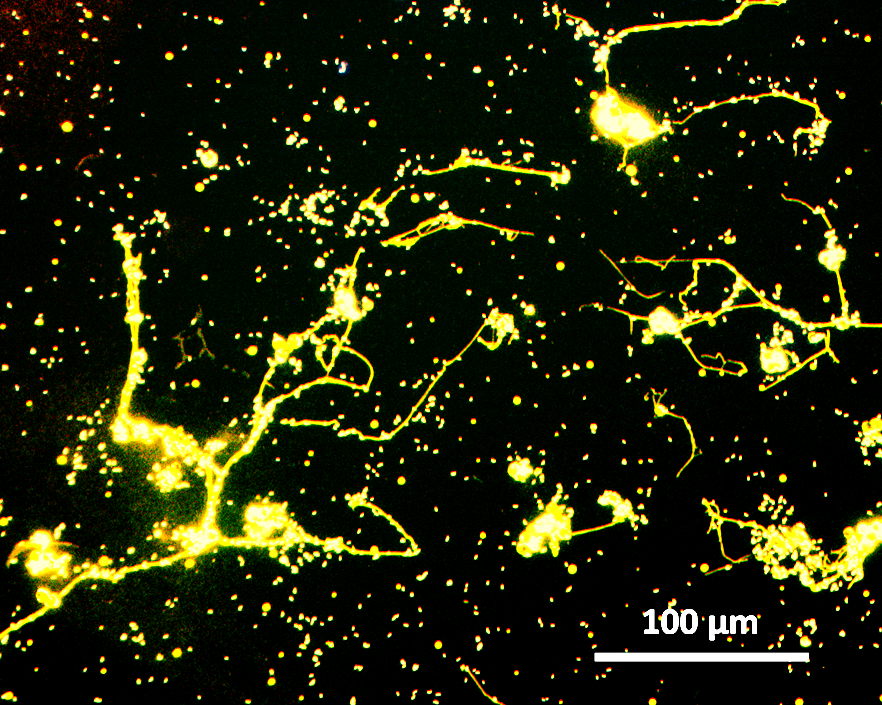


**Fig S8.** Enrichment of cable bacteria after five transfers from the original 1-methylnaphthalene degrading culture hybridized with probe DSB706 specific for *Desulfobulbaceae*.

**
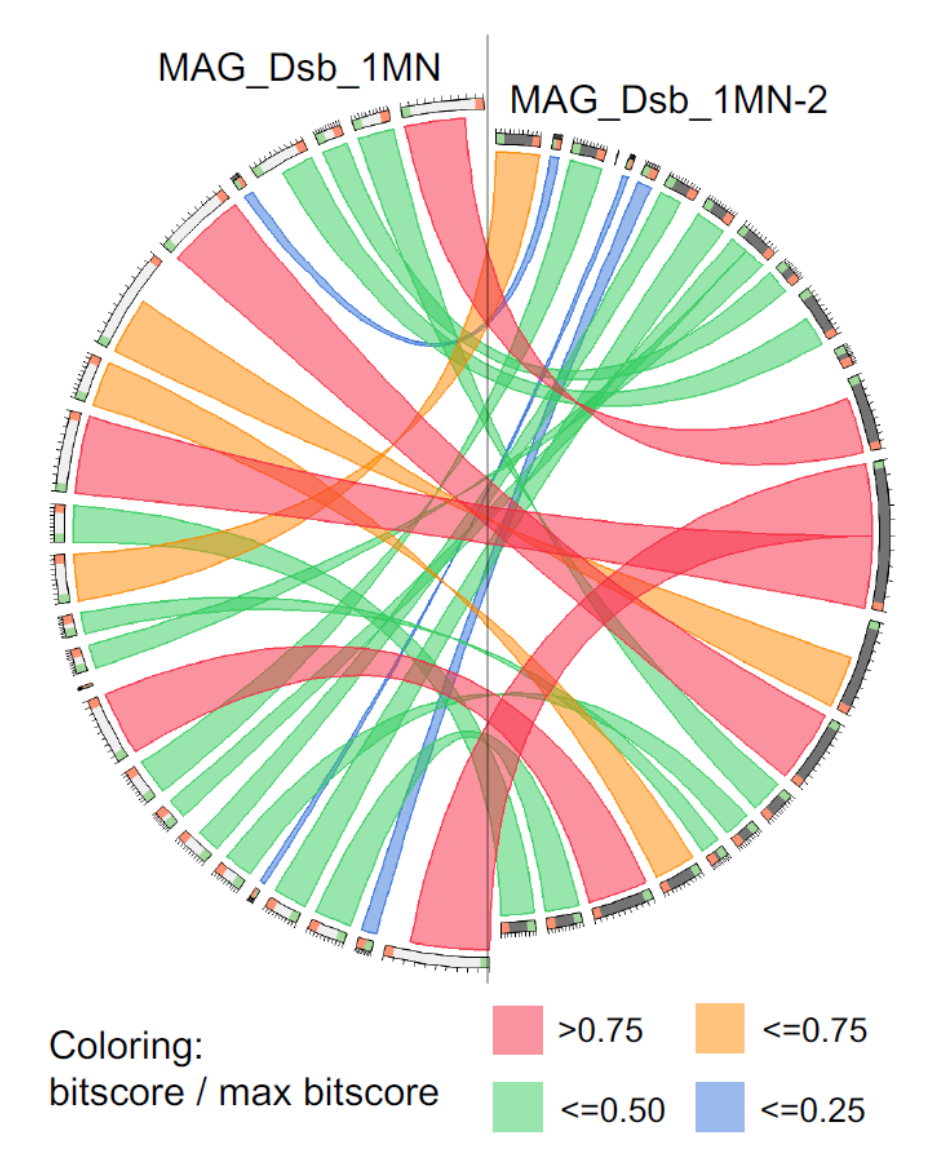
**

**Fig S9.** Whole genome alignments based on blast of all scaffolds of from culture 1MN (MAG_DSB_1MN) and all scaffolds from MAG_DSB_1MN-2 from the cable bacteria enrichment culture. The two genomes show a 99.9% nucleotide identity (0.08% SD, two-way)

**Table S3: Loci and annotations of predicted proteins involved in the central carbon and energy metabolism of the MAG_DSB_1MN deduced from the metagenome of culture 1MN**

|  | **NCBI (nr)** | **Microscope label** | **Gene** | **EC number** | **Product** |
| --- | --- | --- | --- | --- | --- |
| **Glycolysis** | TDB40065 | DSB1MN_v1_10008 | *glk* | 2.7.1.2 | Glucokinase |
|  | TDB40182 | DSB1MN_v1_10158 | *pgi* | 5.3.1.9 | Glucose-6-phosphate isomerase |
|  | TDB40193 | DSB1MN_v1_10170 | *_* | 1.2.1.12 | Glyceraldehyde-3-phosphate dehydrogenase |
|  | TDB40195 | DSB1MN_v1_10172 | *pgk* | 2.7.2.3 | Phosphoglycerate kinase |
|  | TDB40196 | DSB1MN_v1_10173 | *tpiA* | 5.3.1.1 | Triosephosphate isomerase |
|  | TDB38642 | DSB1MN_v1_60032 | *ilvH* | 2.2.1.6 | Acetolactate synthase III, thiamin-dependent, small subunit |
|  | TDB38643 | DSB1MN_v1_60033 | *ilvI* | 2.2.1.6 | Acetolactate synthase III, large subunit |
|  | TDB37329 | DSB1MN_v1_100175 | *fbp* | 3.1.3.11 | Fructose-1,6-bisphosphatase class 1 |
|  | TDB37165 | DSB1MN_v1_120033 | *acs* | 6.2.1.1 | Acetyl-CoA synthetase |
|  | TDB36581 | DSB1MN_v1_140100 | *pki* | 2.7.1.40 | Pyruvate kinase |
|  |  | DSB1MN_v1_150006 | *pfkA* | 2.7.1.11 | ATP-dependent 6-phosphofructokinase |
|  | TDB35996 | DSB1MN_v1_170081 | *eno* | 4.2.1.11 | Enolase |
|  | TDB32057 | DSB1MN_v1_180216 | *adh* | 1.1.1.1 | Long-chain-alcohol dehydrogenase 2 |
|  |  | DSB1MN_v1_180306 | *gap* | 1.2.1.12 | Glyceraldehyde-3-phosphate dehydrogenase |
|  | TDB31033 | DSB1MN_v1_190020 | *apgM* | 5.4.2.12 | putative 2,3-bisphosphoglycerate-independent phosphoglycerate mutase |
|  | TDB31039 | DSB1MN_v1_190030 | *_* | 5.4.2.2 | Phosphoglucomutase |
|  | TDB30932 | DSB1MN_v1_210112 | *_* | 6.2.1.13 | Acetate--CoA ligase [ADP-forming] I |
|  | TDB30969 | DSB1MN_v1_210155 | *gpmI* | 5.4.2.12 | 2,3-bisphosphoglycerate-independent phosphoglycerate mutase |
|  | TDB30221 | DSB1MN_v1_240230 | *ST* | 1.2.7.11 | 2-oxoacid:ferredoxin oxidoreductase 1, subunit beta |
| **TCA cycle** | TDB39194 | DSB1MN_v1_40017 | *acnB* | 4.2.1.3 | Bifunctional aconitate hydratase 2 and 2-methylisocitrate dehydratase |
|  | TDB38642 | DSB1MN_v1_60032 | *ilvH* | 2.2.1.6 | Acetolactate synthase III, thiamin-dependent, small subunit |
|  | TDB38643 | DSB1MN_v1_60033 | *ilvI* | 2.2.1.6 | Acetolactate synthase III, large subunit |
|  | TDB38536 | DSB1MN_v1_70025 | *gltA* | 2.3.3.16 | Citrate synthase 1 |
|  | TDB37208 | DSB1MN_v1_100030 | *accC* | _ | Acetyl-CoA carboxylase, biotin carboxylase subunit |
|  | TDB37218 | DSB1MN_v1_100041 | *mdh* | 1.1.1.37 | Malate dehydrogenase |
|  | TDB32092 | DSB1MN_v1_180256 | *icd* | 1.1.1.42 | Isocitrate dehydrogenase [NADP] |
|  | TDB30760 | DSB1MN_v1_220045 | *fumA* | 4.2.1.2 | Fragment of fumarate hydratase (part 1) |
|  | TDB30761 | DSB1MN_v1_220046 | *fumA* | 4.2.1.2 | Fragment of fumarate hydratase (part 2) |
|  | TDB30221 | DSB1MN_v1_240230 | *ST* | 1.2.7.11 | 2-oxoacid:ferredoxin oxidoreductase 1, subunit beta |
|  | TDB30222 | DSB1MN_v1_240231 | *_* | _ | Pyruvate ferredoxin oxidoreductase |
| **Carbon fixation** | TDB40069 | DSB1MN_v1_10012 | *ackA* | 2.7.2.1 | Acetate kinase A and propionate kinase 2 |
|  | TDB40070 | DSB1MN_v1_10014 | *pta* | 2.3.1.8 | Phosphate acetyltransferase |
|  | TDB40098 | DSB1MN_v1_10046 | *cooS* | 1.2.7.4 | Carbon monoxide dehydrogenase 1 |
|  | TDB39194 | DSB1MN_v1_40017 | *acnB* | 4.2.1.3 | Bifunctional aconitate hydratase 2 and 2-methylisocitrate dehydratase |
|  | TDB38385 | DSB1MN_v1_90062 | *fdhA* | 1.17.1.9 | Formate dehydrogenase subunit alpha |
|  | TDB38396 | DSB1MN_v1_90078 | *acs* | 2.3.1.169 | Carbon monoxide dehydrogenase/acetyl-CoA synthase subunit alpha |
|  | TDB38397 | DSB1MN_v1_90079 | *_* | 1.5.1.20 | Methylenetetrahydrofolate reductase |
|  | TDB38398 | DSB1MN_v1_90080 | *cooS* | 1.2.7.4 | Carbon monoxide dehydrogenase 1 |
|  | TDB38399 | DSB1MN_v1_90081 | *folD* | 1.5.1.5, 3.5.4.9 | Methylenetetrahydrofolate dehydrogenase / Methenyltetrahydrofolate cyclohydrolase |
|  | TDB38401 | DSB1MN_v1_90083 | *fhs* | 6.3.4.3 | Formate--tetrahydrofolate ligase |
|  | TDB37208 | DSB1MN_v1_100030 | *accC* | _ | Acetyl-CoA carboxylase, biotin carboxylase subunit |
|  | TDB37218 | DSB1MN_v1_100041 | *mdh* | 1.1.1.37 | Malate dehydrogenase |
|  | TDB37165 | DSB1MN_v1_120033 | *acs* | 6.2.1.1 | Acetyl-CoA synthetase |
|  | TDB31988 | DSB1MN_v1_180132 | *_* |  | Pyruvate, water dikinase |
|  | TDB32092 | DSB1MN_v1_180256 | *icd* | 1.1.1.42 | Isocitrate dehydrogenase [NADP] |
|  | TDB32133 | DSB1MN_v1_180303 | *_* | _ | Phosphoenolpyruvate synthase |
|  | TDB31038 | DSB1MN_v1_190028 | *pps* | 2.7.9.2 | phosphoenolpyruvate synthase |
|  | TDB30760 | DSB1MN_v1_220045 | *fumA* | 4.2.1.2 | Fumarate hydratase (part 1) |
|  | TDB30761 | DSB1MN_v1_220046 | *fumA* | 4.2.1.2 | Fumarate hydratase (part 2) |
|  | TDB30058 | DSB1MN_v1_240037 | *metF* | 1.5.1.20 | 5,10-methylenetetrahydrofolate reductase |
|  | TDB30129 | DSB1MN_v1_240119 | *_* | 2.7.9.2 | Pyruvate, water dikinase |
|  | TDB30221 | DSB1MN_v1_240230 |  | 1.2.7.11 | 2-oxoacid:ferredoxin oxidoreductase 1, subunit beta |
|  | TDB30222 | DSB1MN_v1_240231 | *_* | _ | Pyruvate ferredoxin oxidoreductase |
| **nitrogen metabolism** | TDB40010 | DSB1MN_v1_20016 | *hcp* | 1.7.99.1 | Hydroxylamine reductase |
|  | TDB39203 | DSB1MN_v1_40027 |  | 1.7.2.2 | Cytochrome c-552 |
|  | TDB37030 | DSB1MN_v1_130008 | *napA* |  | Nitrate reductase |
|  | TDB37032 | DSB1MN_v1_130010 | *nrf* | 1.7.2.2 | Nitrite reductase (cytochrome; ammonia-forming) |
|  | TDB37074 | DSB1MN_v1_130062 | *fprA/nor* |  | Nitric oxide reductase |
|  | TDB30989 | DSB1MN_v1_210021 | *nosL* |  | NosL family protein |
| **sulfur metabolism** | TDB40191 | DSB1MN_v1_10168 |  |  | Sulfite reductase, assimilatory-type |
|  | TDB40168 | DSB1MN_v1_10143 |  |  | Sulfite reductase |
|  | TDB39328 | DSB1MN_v1_30066 | *_* | _ | Heterodisulfide reductase |
|  | TDB39327 | DSB1MN_v1_30065 | *_* | _ | Heterodisulfide reductase subunit A |
|  | TDB39326 | DSB1MN_v1_30064 | *_* | _ | Heterodisulfide reductase subunit A |
|  | TDB39325 | DSB1MN_v1_30062 | *aprA* | 1.8.99.2 | Adenylylsulfate reductase subunit alpha |
|  | TDB39324 | DSB1MN_v1_30061 | *aprB* |  | Adenylylsulfate reductase subunit beta |
|  | TDB39299 | DSB1MN_v1_30030 | *sqr* | 1.8.5.4 | Sulfide-quinone reductase |
|  | TDB39241 | DSB1MN_v1_40069 | *dsrM* |  | Putative electron transfer protein DsrM |
|  | TDB39240 | DSB1MN_v1_40067 | *dsrK* |  | Putative electron transfer protein DsrK |
|  | TDB39239 | DSB1MN_v1_40066 | *dsrJ* |  | Putative electron transfer protein DsrJ |
|  | TDB39238 | DSB1MN_v1_40065 | *dsrO* |  | Putative electron transfer protein DsrO |
|  | TDB39237 | DSB1MN_v1_40064 | *dsrP* |  | Putative electron transfer protein DsrP |
|  | TDB38580 | DSB1MN_v1_70076 | *sat* | 2.7.7.4 | Sulfate adenylyltransferase |
|  | TDB38569 | DSB1MN_v1_70064 | *dsvA/dsrA* | 1.8.99.5 | Sulfite reductase, dissimilatory-type subunit beta |
|  | TDB38568 | DSB1MN_v1_70063 | *dsvB/dsrB* | 1.8.99.5 | Sulfite reductase, dissimilatory-type subunit alpha |
|  | TDB37065 | DSB1MN_v1_130051 | *phsB* |  | Thiosulfate reductase |
|  | TDB37064 | DSB1MN_v1_130050 | *phsA* |  | Thiosulfate reductase |
|  | TDB32015 | DSB1MN_v1_180162 | *dsvC/dsrC* | 1.8.99.5 | Sulfite reductase, dissimilatory-type subunit gamma |
|  | TDB30554 | DSB1MN_v1_230074 |  |  | Rhodanese |
|  | TDB30141 | DSB1MN_v1_240135 | *_* | _ | Sulfurtransferase |
| **F420-nonreducing hydrogenase** | TDB40202 | DSB1MN_v1_10180 | *mvhD* |  | F420-nonreducing hydrogenase |
|  | TDB40203 | DSB1MN_v1_10181 |  |  | 4Fe-4S ferredoxin |
| **Hydrogenase 4** | TDB40323 | DSB1MN_v1_10241 | *hyfC* |  | Hydrogenase |
|  | TDB40254 | DSB1MN_v1_10242 | *hyfE* |  | Hydrogenase |
|  | TDB40255 | DSB1MN_v1_10243 | *hyfF* |  | Hydrogenase |
|  | TDB40256 | DSB1MN_v1_10244 | *hyfG* |  | Hydrogenase |
|  | TDB40257 | DSB1MN_v1_10246 | *hyfI* |  | Hydrogenase |
| **Hydrogenase Ni/Fe** | TDB39380 | DSB1MN_v1_30125 | *hydA* |  | Ni/Fe hydrogenase subunit alpha |
|  | TDB39381 | DSB1MN_v1_30126 | *hydD* |  | NADH:ubiquinone oxidoreductase |
|  | TDB39382 | DSB1MN_v1_30127 | *hydG* |  | Oxidoreductase |
|  | TDB39383 | DSB1MN_v1_30128 | *hydB* |  | Hydrogenase |
| **Hydrogenase 2** | TDB30076 | DSB1MN_v1_240058 | *hybD* |  | Maturation element for hydrogenase 2 |
|  | TDB30077 | DSB1MN_v1_240059 | *hybC* |  | Hydrogenase 2, large subunit |
|  | TDB30078 | DSB1MN_v1_240060 | *hybB* |  | Hydrogenase 2 cytochrome b type component |
|  | TDB30079 | DSB1MN_v1_240061 | *hybA* |  | Hydrogenase 2 4Fe-4S ferredoxin-type component |
|  | TDB30080 | DSB1MN_v1_240062 | *hybO* |  | Hydrogenase 2, small subunit |
| **ATPase (F0F1-type)** | TDB36562 | DSB1MN_v1_140076 | *atpC* |  | ATP synthase epsilon chain |
|  | TDB36563 | DSB1MN_v1_140077 | *atpD* |  | membrane-bound ATP synthase , F1 sector, beta-subunit |
|  | TDB36564 | DSB1MN_v1_140078 | *atpG* |  | ATP synthase gamma chain |
|  | TDB36565 | DSB1MN_v1_140079 | *atpA* |  | F1 sector of membrane-bound ATP synthase, alpha subunit |
|  | TDB36566 | DSB1MN_v1_140080 | *atpH* |  | ATP synthase subunit delta |
|  | TDB36567 | DSB1MN_v1_140081 | *atpF* |  | ATP synthase subunit b |
|  | TDB36568 | DSB1MN_v1_140082 | *atpF* |  | ATP synthase subunit b |
| **Rnf-complex** | TDB36160 | DSB1MN_v1_160090 | *rnfC* |  | Proton-translocating ferredoxin:NAD(+) oxidoreductase complex subunit C |
|  | TDB36161 | DSB1MN_v1_160091 | *rnfD* |  | Proton-translocating ferredoxin:NAD(+) oxidoreductase complex subunit D |
|  | TDB36162 | DSB1MN_v1_160092 | *_* |  | FMN-binding protein |
|  | TDB36163 | DSB1MN_v1_160093 | *rnfE* |  | Proton-translocating ferredoxin:NAD(+) oxidoreductase complex subunit E |
|  | TDB36164 | DSB1MN_v1_160094 | *_* |  | NADH-quinone reductase |
|  | TDB36165 | DSB1MN_v1_160095 | *rnfB* |  | Proton-translocating ferredoxin:NAD(+) oxidoreductase complex subunit B |
|  | TDB36166 | DSB1MN_v1_160097 | *_* |  | Fe-S cluster protein |
| **NADH dehydrogenase** | TDB36222 | DSB1MN_v1_160160 | *nuoA* |  | NADH-quinone oxidoreductase subunit A |
|  | TDB36223 | DSB1MN_v1_160161 | *nuoB* |  | NADH-quinone oxidoreductase subunit B |
|  | TDB36224 | DSB1MN_v1_160162 | *nuoC* |  | NADH-quinone oxidoreductase subunit C |
|  | TDB36225 | DSB1MN_v1_160163 | *nuoD* |  | NADH-quinone oxidoreductase subunit D |
|  | TDB36226 | DSB1MN_v1_160164 | *nuoH* |  | NADH-quinone oxidoreductase subunit NuoH |
|  | TDB36227 | DSB1MN_v1_160165 | *nuoI* |  | NADH-quinone oxidoreductase subunit I |
|  | TDB36228 | DSB1MN_v1_160166 | *nuoJ* |  | NADH-quinone oxidoreductase subunit J |
|  | TDB36229 | DSB1MN_v1_160167 | *nuoK* |  | NADH-quinone oxidoreductase subunit NuoK |
|  | TDB36230 | DSB1MN_v1_160168 | *_* |  | monovalent cation/H+ antiporter subunit D family protein |
|  | TDB36231 | DSB1MN_v1_160170 | *_* |  | hypothetical protein D9V46_07935 |
|  | TDB36232 | DSB1MN_v1_160171 | *nuoM* |  | Na(+)/H(+) antiporter subunit D |
|  | TDB36233 | DSB1MN_v1_160172 | *nuoN* |  | NADH-quinone oxidoreductase subunit N |
| **Cytochrome bd-II oxidase** | TDB37078 | DSB1MN_v1_130067 | *appC* | 1.10.3.- | cytochrome bd-II oxidase, subunit I |
|  | TDB37079 | DSB1MN_v1_130068 | *_* | _ | Cytochrome d ubiquinol oxidase subunit II |
| **Cytochromes** | TDB40107 | DSB1MN_v1_10059 | *_* | _ | Cytochrome C |
|  | TDB40173 | DSB1MN_v1_10148 | *_* | _ | Cytochrome C |
|  | TDB40299 | DSB1MN_v1_10313 | *_* | _ | Cytochrome C |
|  | TDB39203 | DSB1MN_v1_40027 | *nrfA* | 1.7.2.2 | Cytochrome c-552 |
|  | TDB39239 | DSB1MN_v1_40066 | *_* | _ | Cytochrome C |
|  | TDB39241 | DSB1MN_v1_40069 | *hmeC/dsrM* | _ | putative electron transfer protein DsrM |
|  | TDB39250 | DSB1MN_v1_40079 | *dmsE* | _ | DmsE family decaheme c-type cytochrome |
|  | TDB37032 | DSB1MN_v1_130010 | *_* | 1.7.2.2 | Nitrite reductase (cytochrome; ammonia-forming) |
|  | TDB37033 | DSB1MN_v1_130012 | *nrfH* | _ | Cytochrome c nitrite reductase small subunit |
|  | TDB37041 | DSB1MN_v1_130023 | *_* | _ | Chain A iron centre cytochrome C protein |
|  | TDB31918 | DSB1MN_v1_180052 | *ccsB* | _ | C-type cytochrome biogenesis protein CcsB |
|  | TDB31024 | DSB1MN_v1_190009 | *_* | _ | Cytochrome C |
|  | TDB30856 | DSB1MN_v1_210022 | *_* | _ | Cytochrome C biogenesis protein |
|  | TDB30584 | DSB1MN_v1_230005 | *_* | _ | Cytochrome C |
|  | TDB30526 | DSB1MN_v1_230042 | *ppcG* |  | Putative cytochrome C (class III) |
|  | TDB30527 | DSB1MN_v1_230043 | *ppcB* | _ | Cytochrome C (class III) |
|  | TDB30528 | DSB1MN_v1_230044 |  | _ | Cytochrome c biogenesis protein CcsA |
|  | TDB30529 | DSB1MN_v1_230045 | *_* | _ | Cytochrome c biogenesis protein ResB |
|  | TDB30137 | DSB1MN_v1_240128 | *_* | _ | Cytochrome C (class III) |
| **PilA** | ?* | DSB1MN_v1_140048 | *PilA* |  | Type IV pilus assembly protein |
| **Transporter** | TDB40076 | DSB1MN_v1_10020 |  |  | Phosphate/phosphite/phosphonate ABC transporter, periplasmic binding protein |
|  | TDB40137 | DSB1MN_v1_10099 | *ykpA* |  | Uncharacterized ABC transporter ATP-binding protein YkpA |
|  | TDB40172 | DSB1MN_v1_10147 | *feoA* |  | Iron transporter FeoA |
|  | TDB38631 | DSB1MN_v1_60021 |  |  | Cation acetate symporter |
|  | TDB31975 | DSB1MN_v1_180118 |  |  | Sodium:proton antiporter |
|  | TDB32023 | DSB1MN_v1_180174 |  |  | Sodium:calcium antiporter |
|  | TDB37300 | DSB1MN_v1_100142 |  |  | Potassium transporter TrkA |
|  | TDB36252 | DSB1MN_v1_160194 | *amt* |  | Ammonium transporter Amt |

*****not present in the NCBI database. But it is present in “microscope” and also when using our metagenomics pipeline (see materials and methods).

*#DSB1MN_v1_140048|ID:66861477| Type IV pilus assembly protein Deltaproteobacteria bacterium 1MN72D_58_314
MKNQKGFTLVELMIVVAIIGILAAIAIPQFAAYRIRGFNAGAMSDLRNLGTAQEALFADTQGYGSATAAAVLAPAAAVAAPAVITVGPLQAATATVAGAFLHNALGTVGFAVSNGVVTGYSTVVTAAAPVIGTSYVLLAKHTQGDSAYGRDSDSSSMFRATHVAGTALLVADVPAAALGTLELTGTTGAAKNWASMX

**
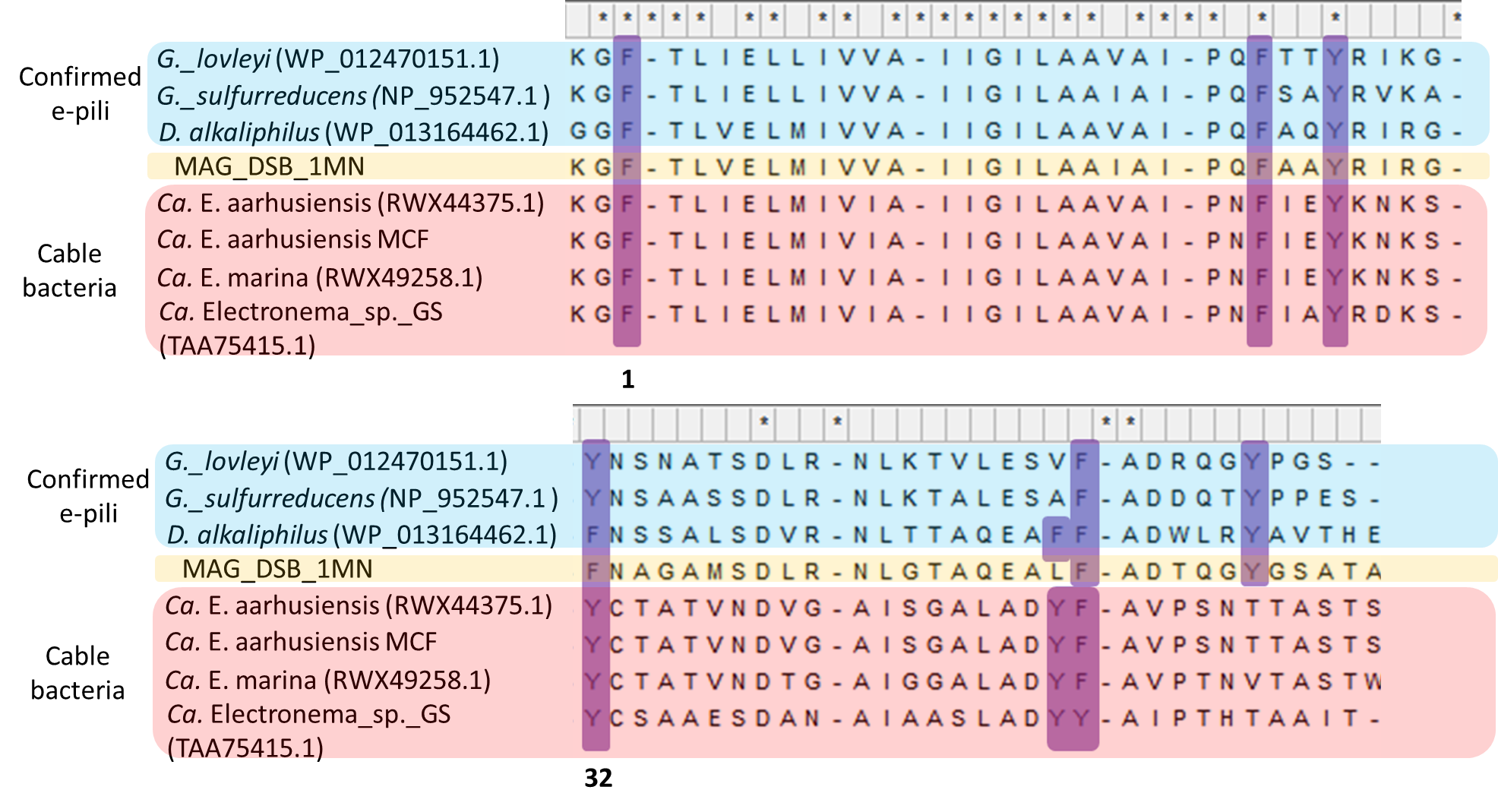
**

**Fig S10. Alignment of PilA amino acid sequences.** Confirmed electrically conductive e-pili (3) (blue shaded) show a highly identical distribution of aromatic amino acids like PilA from MAG DSB 1MN and other cable bacteria. Please not that only the α1-Helix (Positions 1-52) and the first amino acids of the αβ-loop are shown.

References

1. Dick GJ, Andersson AF, Baker BJ, Simmons SL, Thomas BC, Yelton AP, et al. Community-wide analysis of microbial genome sequence signatures. Genome Biol. 2009;10:1.

2. Kearse M, Moir R, Wilson A, Stones-Havas S, Cheung M, Sturrock S, et al. Geneious Basic: an integrated and extendable desktop software platform for the organization and analysis of sequence data. Bioinformatics. 2012;28:1647-9.

3. Walker DJF, Adhikari RY, Holmes DE, Ward JE, Woodard TL, Nevin KP, et al. Electrically conductive pili from pilin genes of phylogenetically diverse microorganisms. The ISME journal. 2017.
